# Regulation of Smoothened Trafficking and Abundance in Hedgehog Signaling

**DOI:** 10.3389/fcell.2022.847844

**Published:** 2022-03-07

**Authors:** Jianhang Jia, Jin Jiang

**Affiliations:** ^1^ Department of Molecular and Cellular Biochemistry, Markey Cancer Center, University of Kentucky College of Medicine, Lexington, KY, United States; ^2^ Department of Molecular Biology, UT Southwestern Medical Center, Dallas, TX, United States

**Keywords:** hedgehog, smoothened, GPCR, ubiquitination, sumoylation, endocytosis, primary cilium, Smurf

## Abstract

The GPCR-family protein Smoothened (Smo) is essential for Hedgehog (Hh) signal transduction in both insects and vertebrates. The regulation of subcellular localization and abundance of Smo is a critical step in Hh signaling. Recent studies have demonstrated that Smo is subjected to ubiquitination mediated by multiple E3 ubiquitin ligases, leading to Smo endocytosis and subsequent degradation through the proteasome- and lysosome-mediated pathways in *Drosophila*. Ubiquitination of Smo also promotes its ciliary exit in mammalian cells. Hh inhibits Smo ubiquitination by blocking E3 ligase recruitment and promoting Smo deubiquitination through the ubiquitin-specific protease 8 (USP8) in *Drosophila*. Inhibition of Smo ubiquitination by Hh promotes Smo cell surface accumulation in *Drosophila* and ciliary accumulation in mammalian cells. Interestingly, Hh also induces sumoylation of Smo in both *Drosophila* and mammalian cells, which promotes Smo cell surface/ciliary accumulation. This review focuses on how ubiquitination and sumoylation regulate Smo intracellular trafficking and abundance and how these processes are regulated by Hh.

## 1 Introduction

**FIGURE 1:**
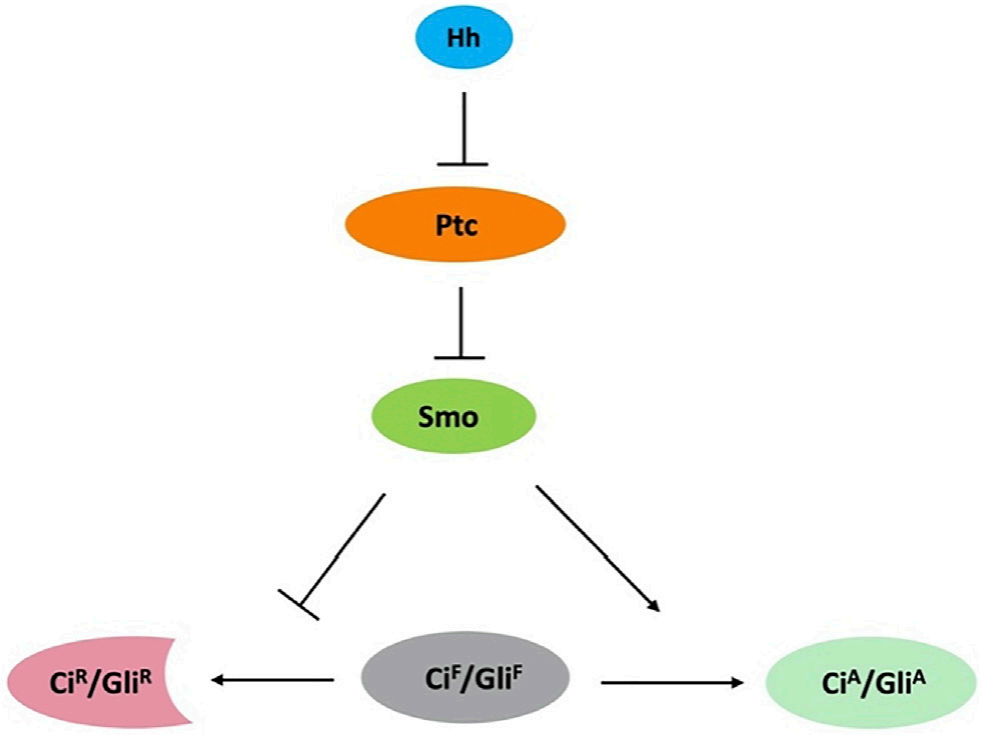
Conserved Hh signaling pathway. Binding of Hh to Ptc releases its inhibition of Smo. Smo inhibits the processing of full-length Ci/Gli (Ci^F^/Gli^F^) into its repressor form (Ci^R^/Gli^R^) and converts Ci^F^/Gli^F^ into its activator form (Ci^A^/Gli^A^).

The Hedgehog (Hh) morphogen controls embryonic development and adult tissue homeostasis in species ranging from insects to mammals (Ingham and McMahon, 2001; Ingham et al., 2011; Jiang and Hui, 2008). Malfunction of Hh signaling has been implicated in many human disorders, including birth defect and cancer (Jiang, 2021; Pasca di Magliano and Hebrok, 2003; Taipale and Beachy, 2001; Villavicencio et al., 2000). Hh signal transduction is largely conserved among species (Figure 1), especially at the plasma membrane, which involves the Hh receptor Patched (Ptc) and coreceptor interference of Hh (Ihog) (Camp et al., 2010; Zheng et al., 2010), and the signal transducer Smoothened (Smo). Smo, a G protein-coupled receptor (GPCR) of the Frizzled-class (class-F), is essential for transducing the Hh signal across the plasma memberane in both insects and vertebrates (Ingham and McMahon, 2001; Jia and Jiang, 2006; Zhao et al., 2007; Jiang and Hui, 2008). Abnormal Smo activation results in basal cell carcinoma (BCC), medulloblastoma, and other types of cancer (Jiang, 2021), making it an attractive therapeutic target.

In *Drosophila*, the Hh signal is transduced at the plasma membrane, where the receptor complex Ptc-Ihog and Smo are located. Hh binding to Ptc-Ihog relieves Smo inhibition by Ptc, resulting in Smo cell surface accumulation and activation, and subsequent activation of the Gli-family of zinc finger transcription factor Cubitus interruptus (Ci) that regulates the expression of Hh target genes. In mammals, Hh signal is transduced at primary cilia where Smo and Gli proteins are accumulated during pathway activation (Bangs and Anderson, 2017). Smo is subjected to post-translational modifications (PTMs). Previous studies have shown that Hh induces phosphorylation of Smo by protein kinase A (PKA), casein kinase 1 (CK1), casein kinase 2 (CK2), and G protein-coupled receptor kinase 2 (GRK2) as well as atypical PKC (aPKC) in *Drosophila* (Chen and Jiang, 2013; Jiang et al., 2014; Li et al., 2014; Jiang and Jia, 2015; Li et al., 2016). Mammalian Smo ciliary localization and activation are also regulated through phosphorylation mediated by CK1 and GRK2 (Chen et al., 2011; Arveseth et al., 2021). Differential phosphorylation and conformational change in Smo mediate the transduction of Hh activity gradient (Jia et al., 2004; Zhao et al., 2007; Chen et al., 2011; Fan et al., 2012). Recent studies have demonstrated that the cell surface accumulation of Smo is regulated by ubiquitination-mediated endocytosis, which is opposed by Smo phosphorylation as well as Smo sumoylation (Zhang and Jiang, 2021). This review focuses on Smo ubiquitination and sumoylation that are the main mechanisms underlying the regulation of Smo trafficking and abundance. We also discuss the similarities and differences in the regulation of Smo between *Drosophila* and mammals where Hh signaling occur in the primary cilium (Bangs and Anderson, 2017).

## 2 Regulation of *Drosophila* Smo Trafficking and Abundance by Multiple E3 Ubiquitin Ligases

Ubiquitination is one of most prevalent PTMs that dictates the fate and function of many cellular proteins (Hershko and Ciechanover, 1998; Popovic et al., 2014). Attachment of ubiquitin to a protein substrate is catalyzed sequentially by ubiquitin activating enzyme (E1), ubiquitin conjugating enzyme (E2), and ubiquitin-protein ligase (E3) that recognizes the protein substrate (Hershko and Ciechanover, 1998). Studies on GPCRs have demonstrated that activation of receptors often stimulates receptor endocytosis mediated by ubiquitination, which plays a role in intracellular signaling in addition to pathway desensitization (Shenoy et al., 2001; Wojcikiewicz, 2004; Irannejad and von Zastrow, 2014). However, unlike other GPCRs whose endocytosis and subsequent degradation is induced upon ligand binding, Smo internalization and cell surface clearance are inhibited in response to Hh stimulation (Denef et al., 2000).

Initial genetic experiments found that inactivation of the Ubiquitin-Activating Enzyme, Uba1, as well as multiple endocytic pathway components in *Drosophila* wing imaginal discs resulted in ectopic cell surface accumulation of Smo in anterior compartment cells that are not exposed to Hh, implying that ubiquitination-mediated endocytosis is involved in removing Smo from the cell surface (Li et al., 2012; Xia et al., 2012). Further biochemical studies revealed that Smo is subjected to both multi-ubiquitination and poly-ubiquitination and is degraded by both proteasome- and lysosome-dependent mechanisms, and that Smo ubiquitination is inhibited by Hh through PKA/CK1-mediated phosphorylation of Smo C-terminal intracellular tail (SmoCT) (Li et al., 2012; Xia et al., 2012).

**FIGURE 2:**
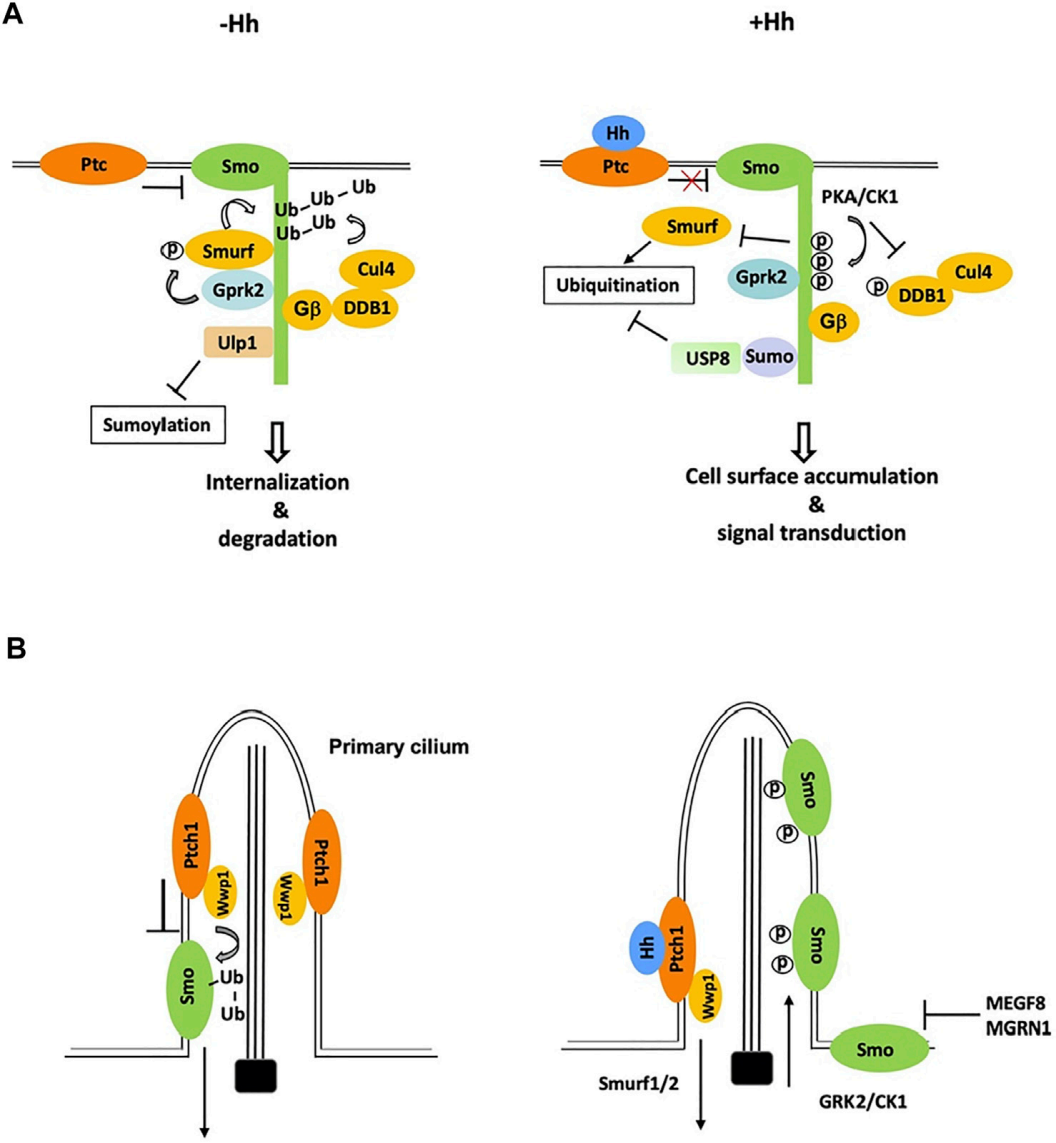
Ubiquitination and sumoylation are involved in the regulation of Smo trafficking and stability. **(A)** In *Drosophila* cells not exposed to Hh, Smo is ubiquitinated by the Smurf family of E3s and other E3s including Cul4-DDB1-Gβ E3 complex, which promotes Smo endocytosis and degradation. Phosphorylation by Gprk2 stimulates the binding of Smurf to Smo. Binding of Ulp1 to Smo blocks its sumoylation. Hh stimulates phosphorylation of Smo and DDB1 by PKA to dissociate Smurf and Cul4-DDB1, respectively, and induces Smo sumoylation to recruit USP8, which blocks Smo ubiquitination, leading to Smo cell surface accumulation. **(B)** In mammalian cells, Wwp1, which is localized in primary cilia through its binding to Patch1, promotes Smo ubiquitination and ciliary exit. Hh inhibits Smo ubiquitination by promoting ciliary exit of Ptch1 and Wwp1, leading to ciliary accumulation of Smo. A membrane-associated E3 complex MEGF8/MGRN1 promotes the degradation of Smo to modulate cells’ sensitivity to Hh.

Much effort has since been devoted to the identification of Smo E3 ligases. The complexity comes from the fact that multiple E3 ligases appear to be involved and therefore, not a single Smo E3 ligase has been identified through the conventional genetic screen. Using a cell-based Smo ubiquitination assay that is sensitive to perturbation, a recent study identified three HECT-domain containing E3 ligases of the Smurf family (also called the Nedd4 family), including Smurf, Nedd4, and Su(dx), whose RNAi reduced Smo ubiquitination in Schneider 2 (S2) cells (Li et al., 2018b). Both loss- and gain-of-function studies in *Drosophila* wing discs suggested that Smurf plays major role while Nedd4 and Su(dx) play minor role in the regulation of Smo cell surface accumulation (Li et al., 2018b). In the absence of Hh, these E3s bind SmoCT through their HECT domains to promote Smo ubiquitination, internalization, and degradation whereas Hh-induced phosphorylation of SmoCT by PKA/CK1 inhibits E3 recruitment and thus Smo ubiquitination, leading to Smo cell surface accumulation (Figure 2A) (Li et al., 2018b).

Interestingly, the binding of Smurf to Smo is promoted by GRK2, which phosphorylates the N-terminal region of Smurf to release an intramolecular autoinhibitory interaction between the N-terminal region and the C-terminally located HECT domain (Li et al., 2018b). This regulatory mechanism could explain why Smo was stabilized in *GRK2* mutant cells (Chen et al., 2010). Release of the Smurf family of E3s from Smo increases their accessibility to Ptc and Hh further stimulates the binding of these E3s to Ptc to promote its ubiquitination and endocytosis (Li et al., 2018b), which accounts for the increased internalization of Ptc in response to Hh (Denef et al., 2000).

A genetic modifier screen identified another HECT-domain and RCC1-like domains (RLDs) containing E3 ligase HERC4 as regulator of Smo ubiquitination and cell surface abundance (Jiang et al., 2019). Like the Smurf family E3s, HERC4 interacted with SmoCT through its HECT domain as well as RCC1-like domains, which is inhibited by Hh-induced phosphorylation of SmoCT by PKA/CK1. Another study showed that mammalian HERC4 is downregulated and negatively correlated with Smo in non-small cell lung cancer (NSCLC) patient samples and that knockdown of HERC4 activated Hh pathway and promoted NSCLC cell proliferation (Sun et al., 2019).

Finally, an *in vivo* RNAi screen for genes whose loss of function resulted in Smo accumulation identified Cullin4 (Cul4) and its binding partner DDB1 as negative regulators of Smo accumulation in wing discs (Li et al., 2018a). Cul4 forms multi-subunit E3 ubiquitin ligase complexes in which DDB1 bridges Cul4 to multiple DDB1-binding WD40 (DWD) proteins that recognize specific substrates (Angers et al., 2006). Biochemical studies revealed that Smo recruits Cul4-DDB1 through multiple β subunits of trimeric G proteins (Gβs) to promote its ubiquitination and turnover, and that Hh signaling recruited the catalytic subunit of PKA (PKAc) to Smo to phosphorylate DDB1, which dissociates Cul4-DDB1 from Gβ and thus inhibits Cul4-mediated Smo ubiquitination (Figure 2A) (Li et al., 2018a).

## 3 Regulation of Mammalian Smo Ciliary Trafficking and Abundance by Ubiquitination

In mammal cells, Hh induces reciprocal trafficking of Ptc and Smo with Ptc moving out and Smo accumulating in primary cilia (Corbit et al., 2005; Rohatgi et al., 2007). Recent studies found that Smo ubiquitination promoted its ciliary exit through IFT27 and BBSome components (Desai et al., 2020; Shinde et al., 2020). Blocking of Smo ubiquitination by pharmacologically inhibiting E1 activity or by mutating two ubiquitin attachment sites in Smo resulted in Smo ciliary accumulation in the absence of Hh whereas fusing a ubiquitin moiety to Smo inhibited its ciliary accumulation in the presence of Hh (Desai et al., 2020). Using CRISPR screen of candidate genes, a subsequent study by the same group identified the HECT domain E3 ligase Wwp1 as essential for Smo ubiquitination and ciliary exit (Lv et al., 2021). Interestingly, Wwp1 is localized in primary cilia through its interaction with Ptc and Ptc-mediated ciliary localization of Wwp1 is critical for promoting Smo ubiquitination and ciliary exit (Lv et al., 2021). Accordingly, Hh-induced ciliary exit of Ptc removes Wwp1 from the primary cilia, which may prevent Smo ubiquitination, leading to Smo ciliary accumulation (Figure 2B). Wwp1 is related to the *Drosophila* Smurf family of E3s and its closest homolog in *Drosophila* is Su(dx), followed by Nedd4 and Smurf. Hence, ubiquitination (Reyes-Turcu et al., 2009) mediated Smo trafficking in *Drosophila* and mammals is controlled by closely related E3 ubiquitin ligases. It will be interesting to determine whether ubiquitination regulates Smo ciliary exit directly by modulating its binding to BBSome and/or other IFT proteins or indirectly by promoting its endocytosis near the ciliary base, a mechanism proposed for ciliary removal of Ptc by Smurf1/2 (Figure 2B) (Yue et al., 2014).

Smo ubiquitination not only regulates its ciliary trafficking but also modulates its overall abundance. Aside from the demonstrated role of HERC4 in the regulation of mammalian Smo ubiquitination and stability in cultured NIH3T3 cells and NSCLC cells (Jiang et al., 2019; Sun et al., 2019), a genome-wide screen for modifiers of Hh pathway activity in cultured cells identified a pair of genes encoding a transmembrane protein MEGF8 and a RING family E3 ligase MGRN1 whose loss-of-function resulted in an increased response to Shh due to elevated Smo levels at the cell surface and primary cilia (Kong et al., 2020). MGRN1and MEGF8 formed a membrane localized E3 ligase complex to catalyze the ubiquitination of Smo, leading to its degradation. Mice homozygous for Megf8 or Mgrn1 mutation exhibited increased Smo abundance and elevated sensitivity to Hh signaling whereas mice heterozygous for either Megf8 or Mgrn1 mutation were normal but double heterozygous embryos exhibited an incompletely penetrant congenital heart defects (CHDs), revealing delicate genetic interactions between Megf8 and Mgrn1 that affect Hh signaling strength through modulating Smo abundance (Kong et al., 2020).

## 4 Regulation of Smo Ubiquitination by Dubs

Ubiquitination is a dynamic process that is reversed by deubiquitinating enzymes (Dubs) (Reyes-Turcu et al., 2009; Komander and Rape, 2012). Several Dubs have been implicated in the regulation of Smo ubiquitination. Loss-of-function of USP8 (also called UBPY) in *Drosophila* wing discs attenuated Smo accumulation in cells exposed to Hh whereas overexpression of USP8 led to ectopic accumulation of Smo and Hh pathway activation (Li et al., 2012; Xia et al., 2012). Consistent with these *in vivo* observations, overexpression of USP8 in S2 cells diminished Smo ubiquitination whereas knockdown of USP8 attenuated Hh-induced reduction of Smo ubiquitination (Li et al., 2012; Xia et al., 2012). Interestingly, the interaction between USP8 and Smo is enhanced by Hh but not by PKA/CK1-mediated phosphorylation of SmoCT, suggesting that Hh regulates USP8 through a PKA/CK1-independent mechanism (Li et al., 2012; Xia et al., 2012).

A genetic modifier screen for genes that modulate the wing phenotype caused by expression of a dominant negative Smo (Smo^DN^) identified UCHL5 (thiol protease class of DUB whose mammalian counterpart is UCH37) as a regulator of Smo ubiquitination and cell surface expression (Zhou et al., 2018; Jiang et al., 2019). UCHL5 binds Smo and promotes its deubiquitination and cell surface accumulation, and the interaction between UCHL5 and Smo is enhanced by Hh (Zhou et al., 2018). Knockdown of UCH37 by RNAi reduced Smo level and Hh pathway activity in NIH3T3 cells, raising the possibility that UCHL5/UCH37 may play a conserved role in modulating Smo ubiquitination and turnover (Zhou et al., 2018).

## 5 Regulation of Smo Trafficking and Abundance by Sumoylation

Similar to protein ubiquitination, sumoylation is another reversible covalent modification that controls many cellular processes involved in protein trafficking and stability control (Geiss-Friedlander and Melchior, 2007). During the sumoylation process, small ubiquitin-related modifier (SUMO) is attached to a target protein, which requires the activation by a SUMO-activation enzyme (E1) and subsequent SUMO transfer by a SUMO-conjugating enzyme (E2). A specific SUMO ligase (E3) is responsible for recognizing the target protein (Johnson, 2004). SUMO molecules can be removed by SUMO-specific isopeptidases (desumoylation enzymes).

Recent studies have linked the SUMO pathway to the regulation of Smo trafficking and abundance (Figure 2A) (Ma et al., 2016; Zhang et al., 2017). *In vivo* RNAi screens in *Drosophila* identified several components of the SUMO pathway whose knockdown modulated the wing phenotypes caused by expressing Smo^DN^ in wing discs. Whereas RNAi of Ubc9, a SUMO-conjugating enzyme E2, PIAS, a SUMO-protein ligase E3, and Smt3, the SUMO isoform in *Drosophila*, enhanced Smo^DN^-induced defects, inactivation of Ubiquitin-like protease 1 (Ulp1) partially suppressed the phenotypes (Ma et al., 2016; Zhang et al., 2017). Consistently, inactivation of Ubc9, PIAS, or Smt3 reduced Hh-induced Smo accumulation and pathway activity in both wing discs and cell cultures (Ma et al., 2016; Zhang et al., 2017). In addition, overexpression of Ulp1 decreased whereas knockdown of Ulp1 increased Smo levels (Ma et al., 2016; Zhang et al., 2017). Hh stimulated sumoylation of Smo mainly on Lys 851, which conforms the sumoylation consensus site (Ma et al., 2016). Substitution of Lys 851 to Arg (Smo^K851R^) greatly diminished Hh-induced Smo sumoylation, reduced Smo stability in cultured cells, and attenuated Hh-induced Smo accumulation and pathway gene expression in wing discs (Ma et al., 2016). The Hh signaling defect caused by the K851R mutation was fully rescued by fusing a SUMO moiety to the C-terminal end of the Smo variant (Ma et al., 2016), demonstrating that sumoylation of Smo at Lys 851 promotes Smo accumulation and Hh pathway activity.

Although binding of UBC9 and PIAS to Smo was undetectable regardless of whether Hh was present or not, Ulp1 interacted with SmoCT in a manner inhibited by Hh signaling, suggesting that Hh stimulates Smo sumoylation mainly by inhibiting the recruitment of the desumoylation enzyme (Ma et al., 2016; Zhang et al., 2017). Like the regulation of USP8-Smo interaction, Ulp1-Smo association and consequently, Smo sumoylation were not regulated by PKA-CK1 mediated phosphorylation of SmoCT (Ma et al., 2016; Zhang et al., 2017), suggesting a link between Smo sumoylation and USP8-mediated Smo deubiquitination. Indeed, Smo sumoylation recruited USP8/UBPY through the Sumo-interacting domain (SIM) present in USP8/UBPY to antagonize Smo ubiquitination, leading to increased cell surface accumulation of Smo (Ma et al., 2016). Interestingly, Kurtz (Krz), the *Drosophila* β-arrestin 2 homolog, blocks Smo sumoylation and prevents its cell surface accumulation (Zhang et al., 2017). Inactivation of Krz decreased the interaction between Smo and Ulp1, suggesting that Krz regulates the sumoylation of Smo through facilitating Smo-Ulp1 interaction (Zhang et al., 2017).

Sumoylation is also involved in the regulation of mammalian Smo trafficking and Shh pathway activity (Ma et al., 2016). In NIH3T3 cells, SUMO-conjugation to Smo was stimulated by Shh but inhibited by overexpression of a mammalian desumoylation enzyme SENP1. Consistent with the notion that sumoylation regulates Shh pathway activity at the level of Smo, overexpression of SENP1 inhibited Shh pathway activity in wild type but not in Sufu mutant MEF cells. Overexpression of SENP1 in NIH3T3 cells inhibited Shh-induced Smo ciliary accumulation and Shh pathway activity, which was reversed by fusion of SUMO to the C-terminal end of Smo (Smo-SUMO). Furthermore, Smo-SUMO exhibited constitutive ciliary accumulation and Shh pathway activity, suggesting that sumoylation of Smo promotes its ciliary localization and activation. However, the sumoylation site(s) on mammalian Smo remains unidentified. In addition, whether sumoylation of Smo promotes its ciliary localization by antagonizing its ubiquitination awaits to be determined. In both *Drosophila* and mammals, how Hh stimulates Smo sumoylation remains a mystery.

## 6 Regulation of Smo by Endocytic Pathway Components

In support of the notion that ubiquitin-mediated endocytic trafficking regulates Smo, several components in the endocytic pathway have been shown to regulate Smo abundance. Inactivation of HGF-regulated tyrosine kinase substrate (Hrs), Tsg101, Avalanche (Avl), Rab5, endosomal sorting complex required for transport complex-II and III (ESCRT-II and ESCRT-III) in *Drosophila* wing discs resulted in accumulation of Smo in the absence of Hh (Li et al., 2012; Fan et al., 2013; Yang et al., 2013; Jiang et al., 2018). Hrs interacts with Smo and promotes its ubiquitination (Fan et al., 2013). In addition, the ESCRT-III core subunits, VPS32 (also known as Shrub in *Drosophila*, SNF7 in yeast, and CHMP4 in mammal) and VPS20 (CHMP6 in mammals) regulate Smo stability intracellularly (Jiang et al., 2018). Surprisingly, Smo is accumulated in phosphorylated and active forms in cells defective in ESCRT-III function, which does not rely on Hh stimulation, indicating that Smo can exhibit Hh-independent activity when accumulated at high levels in a specific intracellular compartment. Furthermore, a Krz-mediated pathway, operating in parallel to endocytosis, directs Smo to the ESCRT-III/multivesicular body (MVB), leading to the high accumulation and activation of Smo when ESCRT-III function is inactivated (Jiang et al., 2018). Of note, the ESCRT machinery has also been shown to regulate the secretion and long-range Hh signaling (Matusek et al., 2014), suggesting that the ESCRT machinery regulates Hh signaling in both signal sending and receiving cells.

## 7 Conclusion and Perspectives

Many scaffolding proteins and enzymes are involved in Smo ubiquitination, sumoylation, and intracellular trafficking, as discussed in this review; however, how these events are sophisticatedly controlled by Hh signaling has not been fully understood. Because vertebrate Hh signaling occurs in primary cilia and may utilize different mechanisms to regulate Smo, further studies in vertebrate systems are needed to explore the link between the various enzymes involved in ubiquitination and sumoylation and their possible involvement in human diseases. For example, while phosphorylation of *Drosophila* Smo (dSmo) by PKA inhibits its ubiquitination, mammalian Smo (mSmo) is not phosphorylated by PKA and whether phosphorylation of mSmo by other kinases such as CK1 and GRK2 regulates its ubiquitination remains to be determined. How mSmo sumoylation is stimulated by Hh and whether mSmo sumoylation also inhibits its ubiquitination remain to be explored. Although targeted expression of a UbK63-specific Dub could promote mSmo ciliary accumulation (Shinde et al., 2020; Lv et al., 2021), whether Shh stimulates binding of a Dub(s) to antagonize mSmo ubiquitination remains to be determined. It is interesting to note that Hh stimulates the binding of PKA catalytic subunit (PKAc) to SmoCT in both *Drosophila* and mammalian systems (Li et al., 2014; Ranieri et al., 2014; Arveseth et al., 2021). While binding of PKAc to dSmo is thought to promote dSmo phosphorylation and sequester PKAc away Ci, binding of PKAc to a PKI motif in vertebrate SmoCT has been shown to sequester PKAc away from Gli2 and Gli3 and thus inhibit Gli phosphorylation and processing (Happ et al., 2021). It would be interesting to determine whether a similar PKI motif is present in dSmo and contributes its inhibition of Ci phosphorylation.

The mechanisms underlying Smo regulation are the subject of intense interest because aberrant Smo activation contributes to many type of human cancer including basal cell carcinoma and medulloblastoma (Xie et al., 1998; Yang et al., 2010; Jiang, 2021), making Smo an attractive therapeutic target as exemplified by the U.S. FDA approved drugs, such as vismodegib, sonidegib, and glasdegib for the treatment of cancers known to be driven by Smo activation (Guha, 2012; Sekulic and Von Hoff, 2016; Pietrobono and Stecca, 2018; Bohl et al., 2019). The regulation of Smo intracellular trafficking and abundance is a major mechanism controlling Hh pathway activity. Future studies of additional players involved in the regulation of Smo ubiquitination, sumoylation, and trafficking, as well as elucidating their functional and regulatory relationships, may provide new avenues for developing novel cancer therapeutics.
